# Chemokine receptors differentially expressed by race category and molecular subtype in the breast cancer TCGA cohort

**DOI:** 10.1038/s41598-022-14734-5

**Published:** 2022-06-26

**Authors:** Elissa D. Vazquez, Xiangyi Fang, Lauren A. Levesque, Mike Huynh, Citlali Venegas, Nhien Lu, Nicole Salazar

**Affiliations:** Department of Biology, San Francisco State University, San Francisco, CA 94132 USA

**Keywords:** Breast cancer, Cancer

## Abstract

Racial disparities in mortality due to metastasis remain significant among breast cancer patients. Chemokine receptors contribute to breast tumors and metastatic outcome. We explored for significant differences in chemokine receptor expression in breast tumors from Black, Asian, and White patients in The Cancer Genome Atlas. We show that despite sharing the same molecular subtype, expression of the chemokine receptors ACKR1, CCR3, CCR6, CCRL1, CCRL2, CXCR1, CXCR2, CXCR4, CXCR6, and CXC3CR1 was significantly different depending on racial group. For patients with triple negative breast cancer, CCR3 was higher in Black versus White and CCRL2 was higher in Asian versus White. In luminal A tumors, ACKR1 was lower in Asian versus White, CCR3 was higher in Black versus White, and CCR6 and CXC3CR1 were lower in Black versus White. In luminal B tumors, CCRL2 was lower in Black versus White, CXCR1 and CXC3CR1 were lower in Asian versus White, and CXCR2 was lower in Black and Asian versus White. In HER2 enriched tumors, CCR3 was higher in Black versus White and CXCR4 lower in Asian versus White. CCR3, CCR6, and CXCR6 associated with worse patient survival. These findings can inform improved treatment strategies to decrease racial disparities in breast cancer burden.

## Introduction

An estimated 43,250 women and 530 men will die from breast cancer in the year 2022 in the United States and approximately 685,000 women will die worldwide^1,2^. Racial disparities in mortality due to metastasis remain significant among breast cancer patients^3–6^. Black women are more likely to die from more aggressive and metastatic breast cancer than other racial groups^6,7^. Despite having the lowest incidence of breast cancer rates across racial groups, the rates have increased in the last decade for Asian and Pacific Islander women in the United States^6,8^. The concurring summary from a robust emerging body of literature is that racial disparities observed in cancer progression and outcome are a combination of social, environmental, ancestral and biological factors^9–12^. Biological factors refer to the genetic, epigenetic, cellular, and molecular level changes associated with disease development and progression. Biological factors are apparent when analyzing the cells within the context of their unique tissue microenvironment.

Chemokines and their receptors are proteins that contribute significantly to the tumor microenvironment^10,13–15^. Chemokine receptors are essential to the migratory pattern and positioning of cells and are required for normal processes, including immune cell trafficking and inflammation^16,17^. Chemokine receptor regulation plays an important role in the tumor microenvironment, being expressed not only by cancer cells but also by cells that compose the tumor stroma, including immune cells, structural cells (i.e. fibroblasts and pericytes), and endothelial cells. They also play an important role in tumor development and metastatic outcome^18–20^. Recent studies identified genes associated with breast cancer progression using weighted gene co-expression network analysis (WGCNA) using The Cancer Genome Atlas (TCGA) database^21–26^. In other studies, Keenan et al. and Huo et al. compared the genomic landscape between Black and White women in the TCGA breast cancer cohort and demonstrated significant differences in tumor biology that can result in survival disparities^12,27^. Here, we used breast cancer tissue RNA sequencing data from the TCGA breast cancer cohort to assess the expression levels for the chemokine receptors as described by the IUPHAR^17^ in breast tumors across molecular subtypes from patients of different racial categories. These findings provide a basis to study connections between chemokine receptors and breast cancer molecular subtype and their contribution to the breast tumor microenvironment in patients from different racial categories.

## Results

### WGCNA and module-trait relationships

To identify if chemokine receptor genes in breast cancer are associated with specific clinical traits, we performed WGCNA to construct modules of co-expressed genes from breast invasive carcinoma bulk RNA sequencing data from the TCGA from all available patient samples (Supplementary Fig. 1A). We identified race as the trait with strongest clinical module-trait relationship (Supplementary Fig. 1B), when comparing available clinical traits from the GDC TCGA data. Next, we defined a variable as “sample type” to determine if the difference between normal and tumor samples could explain major differences in clinical module-trait relationships. Supplementary Fig. 1C shows the modified module-trait relationship from the combined normal and tumor samples with clinical traits, where the trait that made the most significant difference/strongly associated with modules was “sample type.” Therefore, including both normal and tumor samples together in the same WGCNA analysis showed disease status as the major driver of observed differences. As the initial analysis indicated that race showed the strongest module-trait relationships (Supplementary Fig. 1B), we next asked whether these relationships could be explained not by race but rather due to molecular subtypes, as racial groups differ in their breast tumor molecular subtype incidence^8,28,29^ and included the hormone receptor status^30^ as part of the clinical trait relationship analysis, which showed that after sample type, hormone receptor status had the strongest module-trait relationships (Supplementary Fig. 1C).

**Figure 1:**
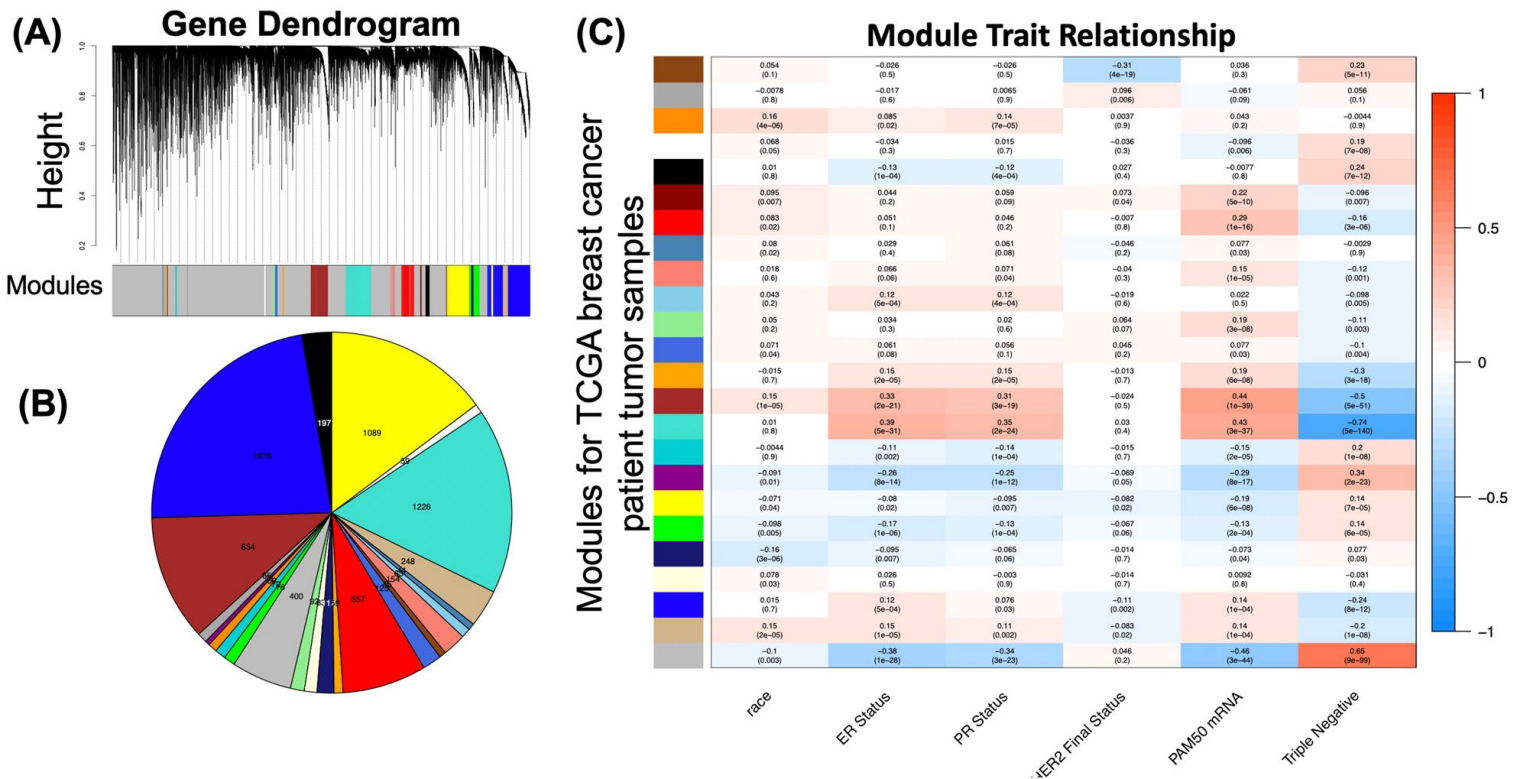
Network construction and module detection for Weighted Gene Co-expression Network Analysis (WGCNA) of Breast Invasive Carcinoma RNA-seq samples from TCGA database. (**A**) Gene dendrogram of clustered gene dissimilarity, based on consensus topological overlap, with corresponding gene module colors of the TCGA breast invasive carcinoma RNA-seq dataset from n = 824 tumor patient samples, each with 20,253 genes analyzed in one block. (**B**) Number of genes in each module identified from WGCNA. The modules containing the greatest number of genes are the blue, turquoise, yellow, and brown modules. (**C**) Module-trait associations for tumor only samples (excluding normal samples) from the cohort. The corresponding *p*-value for the correlation of the module with the clinical parameter is in parenthesis. The grid is color-coded by correlation according to the color bar.

We repeated the WGCNA and module-trait relationship analysis, analyzing only tumor samples by removing the normal samples available from the breast TCGA cohort. We identified 23 co-expressed modules in the tumor only WGCNA (Fig. 1A,B). Using the identified modules from the WGCNA results, we generated the module-trait relationship analysis to identify which modules/gene expression changes were associated with the specific clinical traits of interest. Figure 1C shows the module-trait relationship analysis between tumor samples and clinical traits, where the traits most strongly associated with modules are molecular subtypes and PAM50 status, which are associated to breast tumor progression.

### Differential gene expression analysis

We performed differential gene expression analysis comparing Black versus White and Asian versus White because these three groups had the highest number of patient samples available from the TCGA. Hispanic ethnicity could not be associated with a race group and Native American only had 1 sample, therefore could not be used for group analyses. The differentially expressed genes (DEGs) between all the available Black and White patients in the TCGA cohort are in Supplementary-Table 1. The DEGs between all the available Asian and White patients in the TCGA cohort are in Supplementary-Table 2). We searched for known chemokine receptors to identify which were differentially expressed between racial categories and labeled them in Table 1, which shows ACKR1, CCRL2, and CXCR2 are the only common chemokine receptor DEGs for both Black versus White and Asian versus White samples. CCR3, and ACKR4/CCRL1 were also differentially expressed between Black versus White samples. CCR6, CXCR1, CCRL2, CXCR4 and CXCR6 were differentially expressed between Asian versus White samples.

**Table 1:** Significant (*p* value < 0.05) DEG for Chemokine Receptors for two analysis, either all samples in TCGA Breast cohort or for the tumor samples only.

	log2FoldChange	padj
**For all (normal and tumor) samples in TCGA breast cohort**		
Black versus White		
**ACKR1/DARC**	− 1.153	0.004
CCR3	0.687	0.016
CCRL1	− 0.641	0.014
**CCRL2**	− 0.412	0.023
**CXCR2**	− 0.413	0.007
Asian versus White		
**ACKR1/DARC**	− 1.594	0.000
CCR6	− 0.471	0.012
**CCRL2**	0.599	0.001
CXCR1	− 1.062	0.024
**CXCR2**	− 0.417	0.011
CXCR4	− 0.448	0.046
CXCR6	0.667	0.026
**For tumor samples in TCGA breast cohort**		
Black versus White		
**ACKR1/DARC**	− 0.450	0.048
CCR3	0.661	0.006
CX3CR1	− 0.416	0.046
Asian versus White		
**ACKR1/DARC**	− 1.064	0.004
CCR6	− 0.476	0.005
CCRL2	0.617	0.000
CXCR4	− 0.406	0.026

Bolded receptors names indicate those common in both Black versus White and Asian versus White comparisons. Full list of DEG in Supplementary tables provided.

The DEGs for tumor only samples between Black versus White patients in the TCGA cohort are in Supplementary Fig. 2A and Supplementary-Table 3 and for Asian versus White patients in Supplementary Fig. 2B and Supplementary-Table 4). The top differentially expressed genes in tumors between Black and White patients after adjusting for covariate effects, were RPL29P2 and CRYBB2. The top differentially expressed genes in tumors between Asian and White patients, after adjusting for covariates, were CHGB and LEP (supplementary Tables 2 and 4). Table 1 shows ACKR1/DARC was the only common chemokine receptor DEG between Black versus White and Asian versus White patient tumor samples. CCR3 and CX3CR1 were differentially expressed between Black versus White, and CCR6, CCRL2, and CXCR4 between Asian versus White breast tumors.

Differences between normal and tumor tissues have been well described^31,32^. Significant differences in gene expression in normal versus tumor samples within the breast cancer TCGA cohort have been reported before^31^. However, when we queried for differentially expressed genes in the limited available normal adjacent versus tumor tissues (Supplementary-Tables 5 and 6), we identified only the chemokine receptors CXCR2, CXCR6, and CX3CR1 to be commonly differentially expressed in normal versus tumor samples for both Black and White patients. We were unable to do normal versus tumor analysis for the Asian group due to the lack of normal tissue samples for Asian patients in the breast cancer TCGA.

### Gene expression analysis

**Figure 2:**
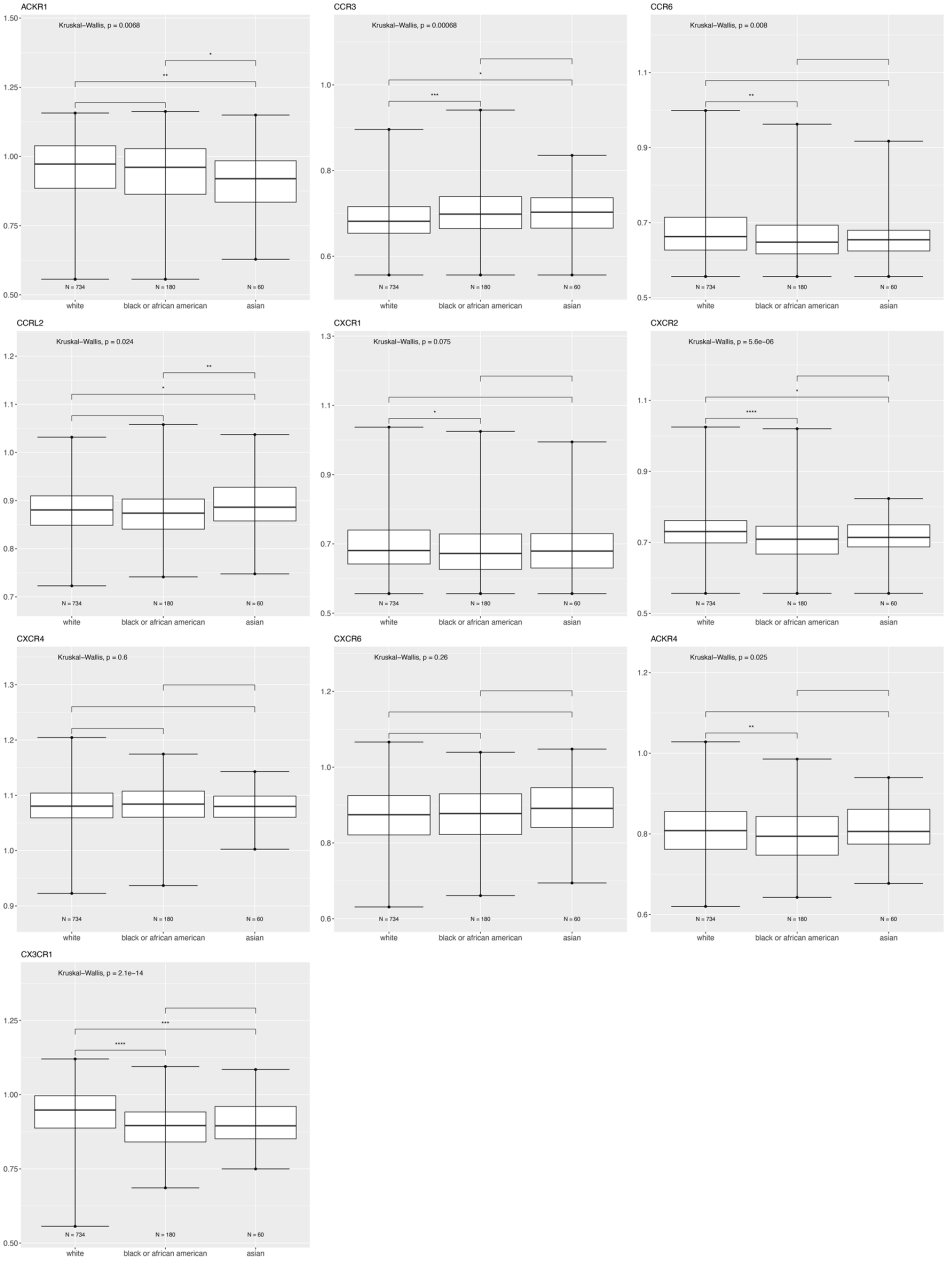
Differentially expressed chemokine receptor genes based on race using the breast cancer TCGA data for tumor samples only. Box and whisker plots represent minimum expression at the bottom whisker, maximum at the top whisker and median at the middle line with a Log10 axis scale showing gene expression of the chemokine receptors. Statistical significance was determined with global significance for ANOVA with Kruskal Wallis.

We plotted the tumor gene expression data to illustrate how the chemokine receptor genes are expressed across race and tumor molecular subtype. Figure 2 shows ACKR1, CCR3, CCR6, CCRL2, CXCR2, and CX3CR1 expression is significantly different based on race category (White, Black or Asian), using the breast cancer TCGA data for tumor samples.

**Figure 3:**
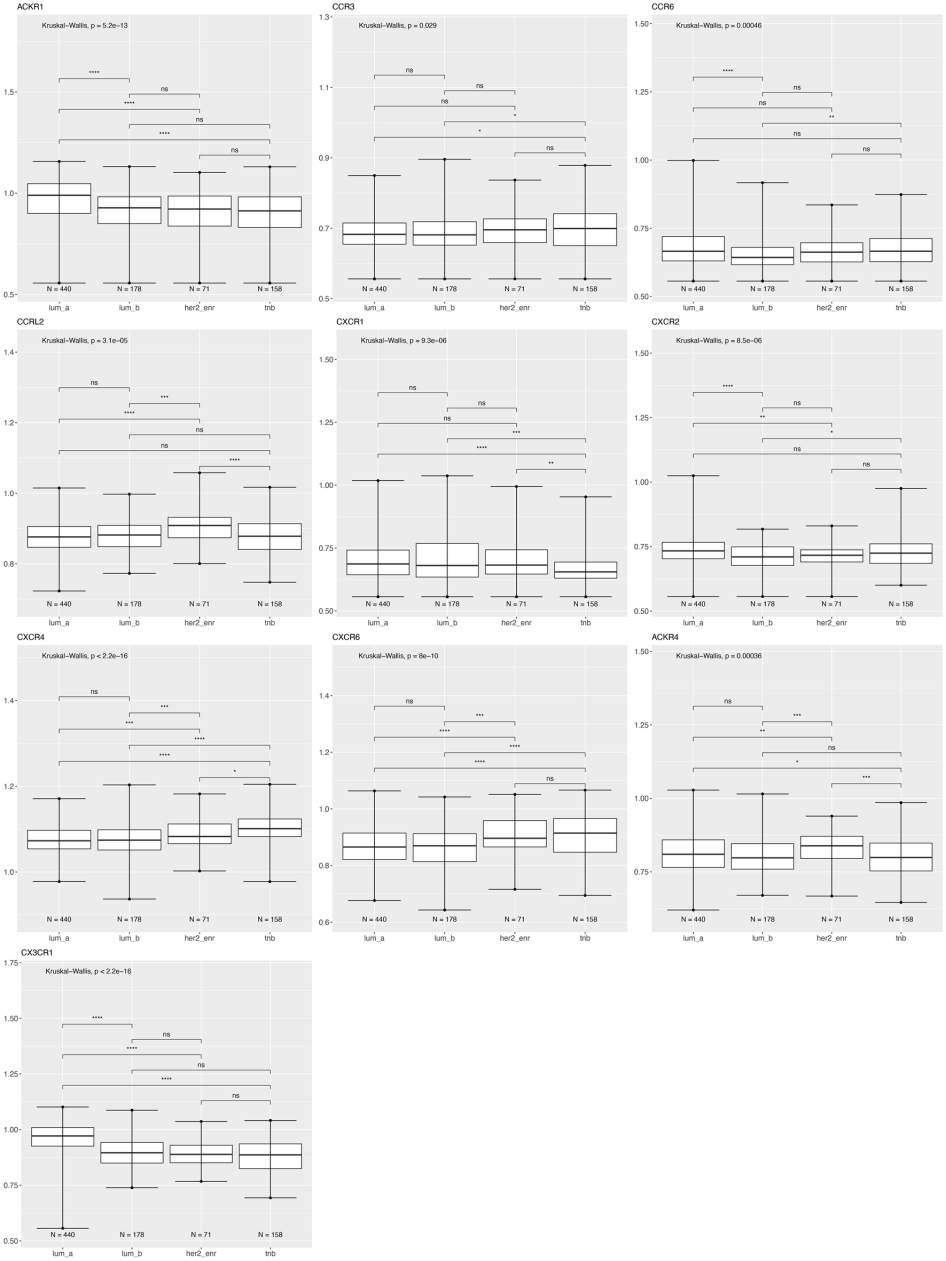
Differentially expressed chemokine receptor gene expression based on molecular subtype using the breast cancer TCGA data for tumor samples only. Box and whisker plots represent minimum expression at the bottom whisker, maximum at the top whisker and median at the middle line with a Log10 axis scale showing gene expression of the chemokine receptors. Statistical significance was determined with global significance for ANOVA with Kruskal Wallis.

Since breast tumor molecular subtype (Luminal A or B, HER2 enriched, and triple negative), is a well-established determinant of differences in patient outcome^33^, we analyzed receptor expression within molecular subtypes across racial categories. Figure 3 shows ACKR1, CCR3, CCR6, ACKR4/CCRL1, CCRL2, CXCR1, CXCR2, CXCR4, CXCR6, and CX3CR1 expression is significantly different based on breast cancer molecular subtypes.

**Figure 4:**
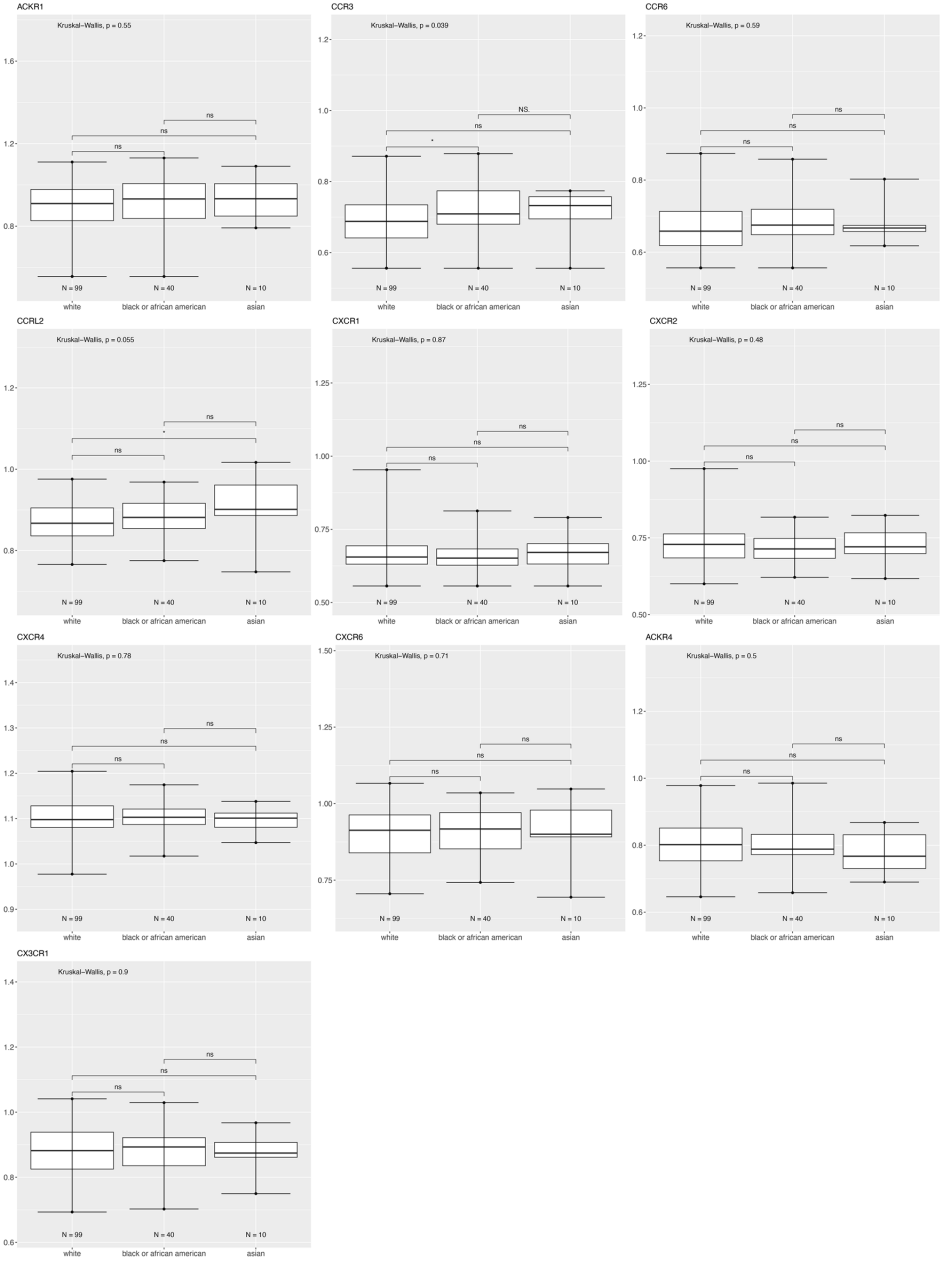
Differentially expressed chemokine receptor gene expression based on race within triple negative subtype using the breast cancer TCGA data for tumor samples only. Box and whisker plots represent minimum expression at the bottom whisker, maximum at the top whisker and median at the middle line with a Log10 axis scale showing gene expression of the chemokine receptors. Statistical significance was determined with global significance for ANOVA with Kruskal Wallis.

Figure 4 shows that for patients with triple negative breast cancer, CCR3 is significantly higher in Black versus White patients while CCRL2 is higher in Asian versus White patients.

**Figure 5:**
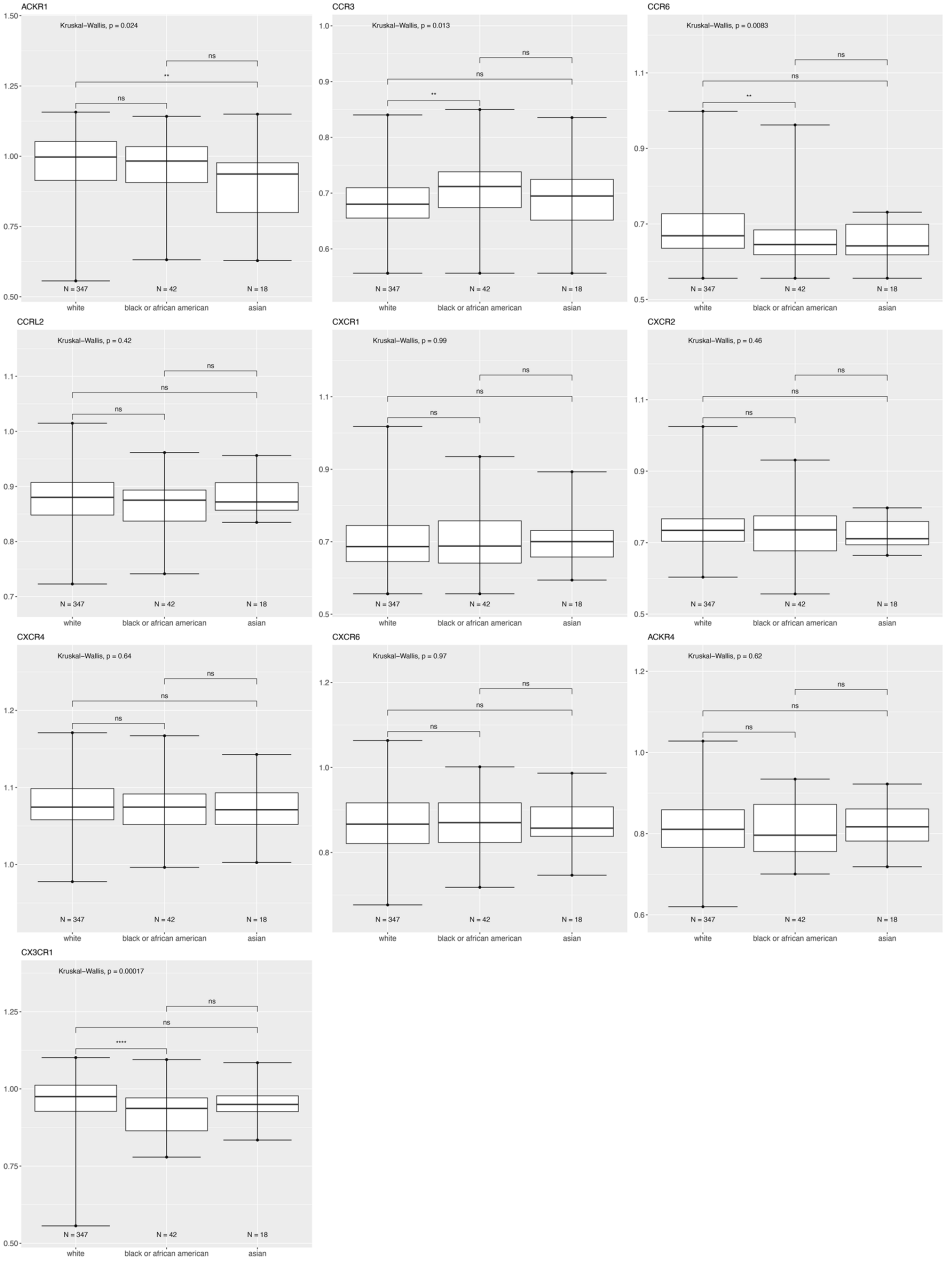
Differentially expressed chemokine receptor gene expression based on race within Luminal A subtype using the breast cancer TCGA data for tumor samples only. Box and whisker plots represent minimum expression at the bottom whisker, maximum at the top whisker and median at the middle line with a Log10 axis scale showing gene expression of the chemokine receptors. Statistical significance was determined with global significance for ANOVA with Kruskal Wallis.

Figure 5 shows that for patients with luminal A breast cancer, expression of ACKR1 is lower in Asian than White, CCR3 is higher in Black versus White, and CCR6 and CXC3CR1 are lower in Black versus White.

**Figure 6:**
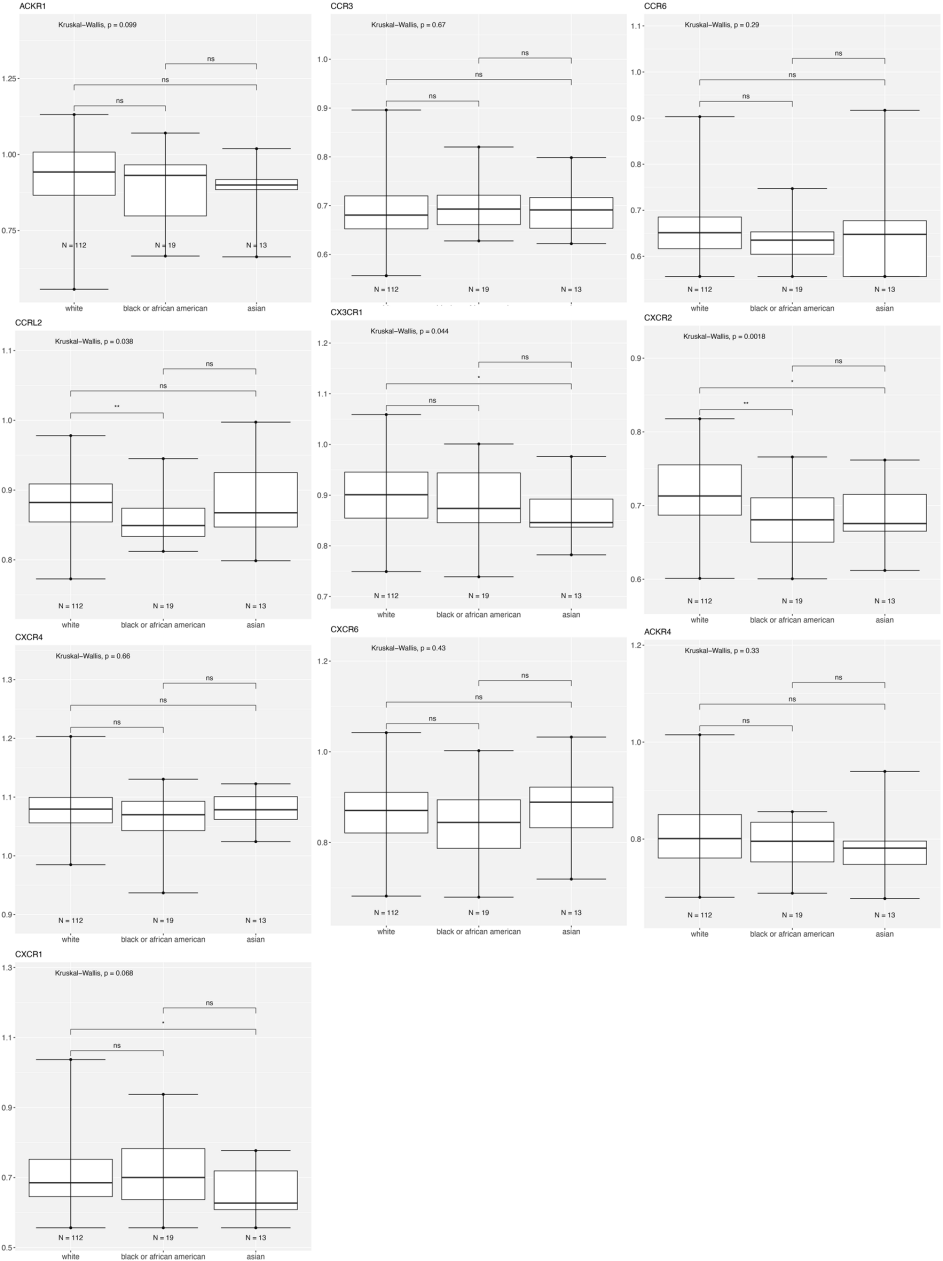
Differentially expressed chemokine receptor gene expression based on race within Luminal B subtype using the breast cancer TCGA data for tumor samples only. Box and whisker plots represent minimum expression at the bottom whisker, maximum at the top whisker and median at the middle line with a Log10 axis scale showing gene expression of the chemokine receptors. Statistical significance was determined with global significance for ANOVA with Kruskal Wallis.

Figure 6 shows that in patients with luminal B breast cancer, expression of CCRL2 is lower in Black versus White, CXCR1 and CX3CR1 are lower in Asian versus White, and CXCR2 is lower in both Black and Asian versus White.

**Figure 7:**
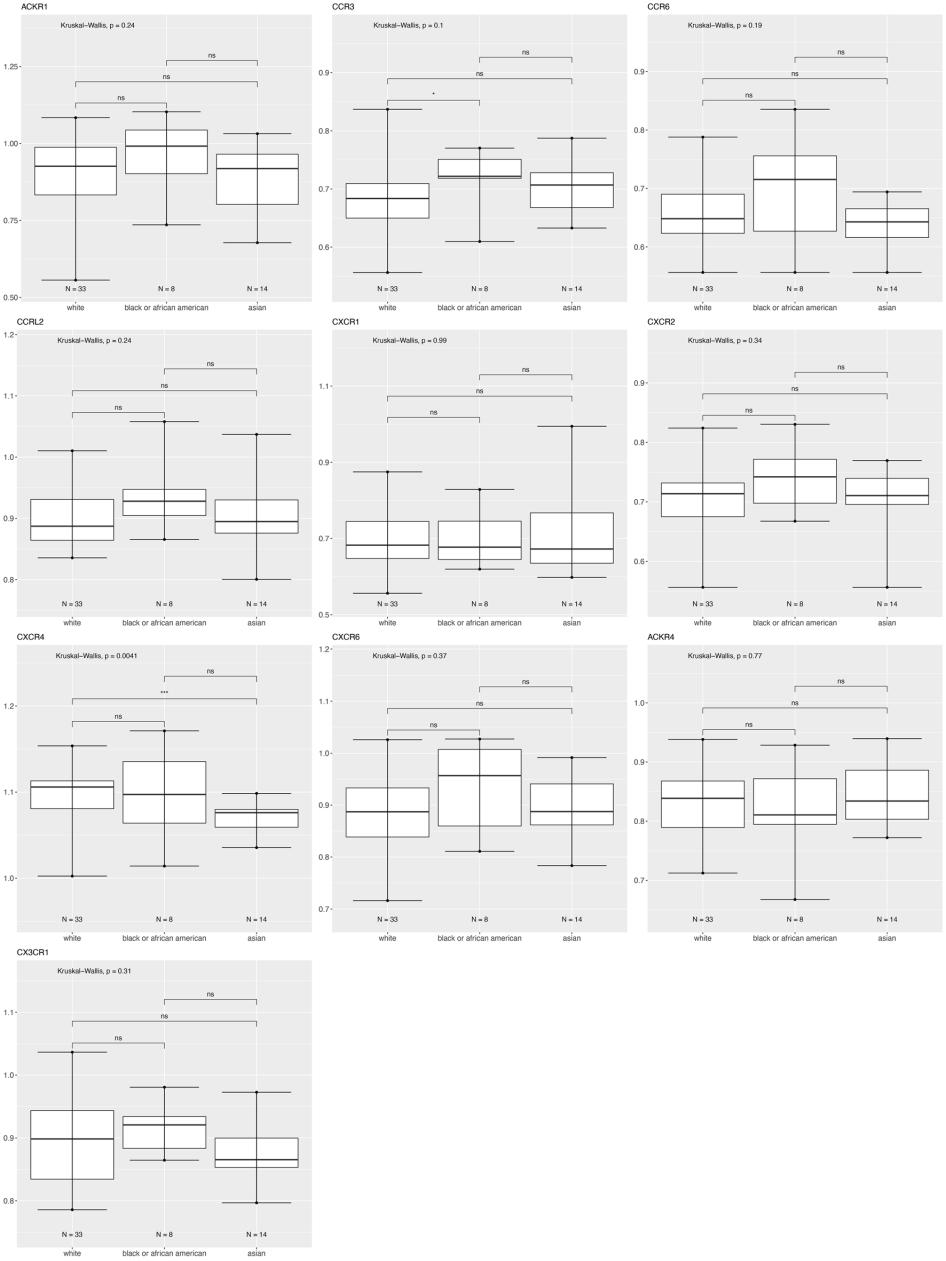
Differentially expressed chemokine receptor gene expression based on race within HER2 subtype using the breast cancer TCGA data for tumor samples only. Box and whisker plots represent minimum expression at the bottom whisker, maximum at the top whisker and median at the middle line with a Log10 axis scale showing gene expression of the chemokine receptors. Statistical significance was determined with global significance for ANOVA with Kruskal Wallis.

Figure 7 shows that for patients with HER2 enriched tumors there is higher CCR3 in Black versus White, and lower CXCR4 in Asian versus White breast cancer patients.

### Survival analysis

**Figure 8:**
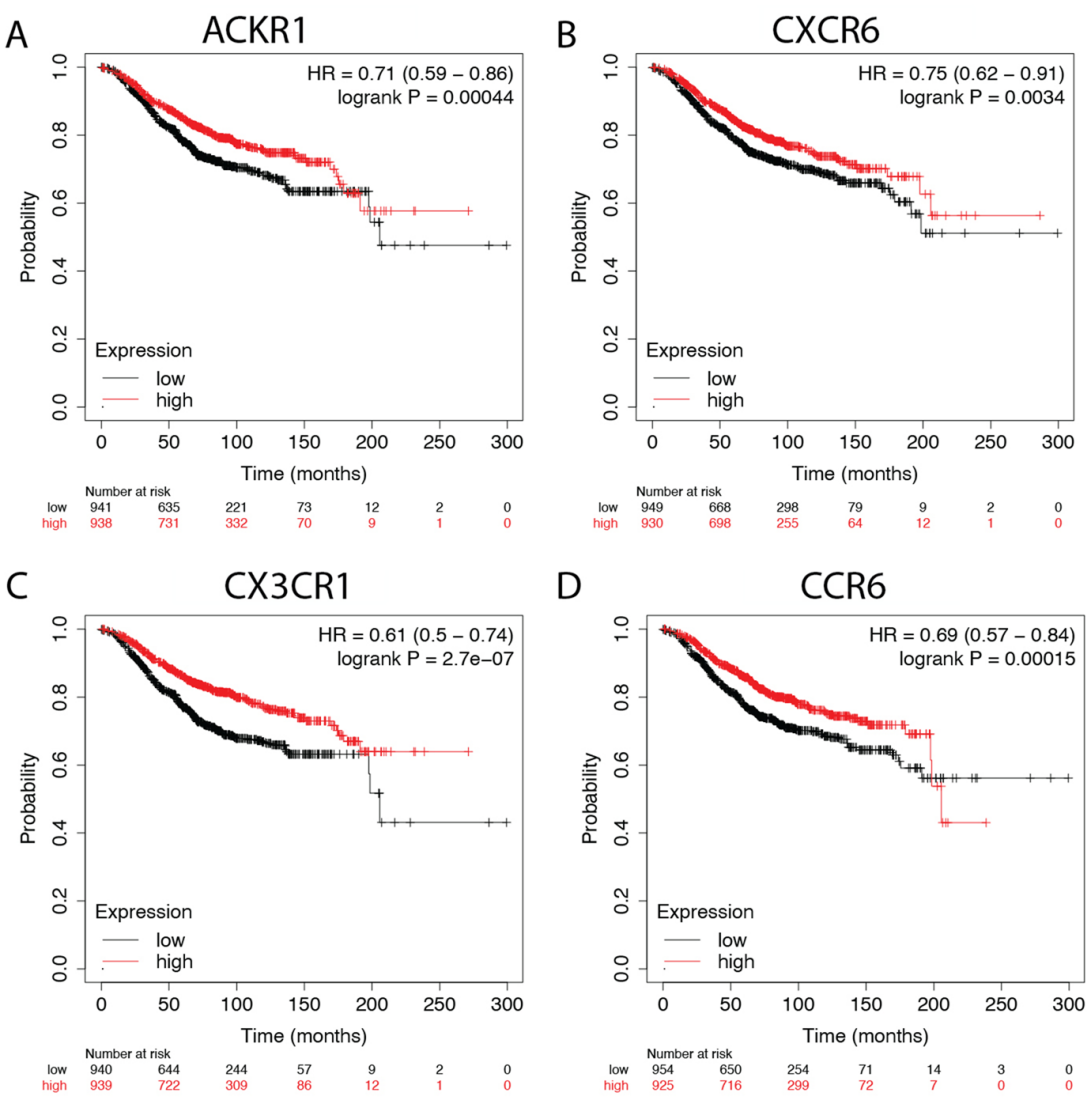
Overall survival rate of breast cancer patients tumor samples. Kaplan–Meier curves of overall survival based on KMplotter mRNA gene chip expression of chemokine receptors in breast cancer patients. Red line represents higher expression and black line represents lower expression. Patients with a high expression of the genes for (**A**) ACKR1, (**B**) CXCR6, and (**C**) CX3CR1 have a higher survival rate than those who have a low expression of the gene. (**D**) CCR6 low expression had better survival only at later time points.

Ten chemokine receptors (CXCR1, CXCR2, CXCR4, CXCR6, CCR3, CCR6, ACKR4/CCRL1, CCLR2, CX3CR1, and ACKR1/DARC) were identified as differentially expressed from the DEG analysis between Black, White and Asian patient normal and tumor samples. We used overall survival (OS) for breast cancer tumors in TCGA and in the GEO repository to determine if receptor expression correlated with survival. Figure 8 shows Kaplan–Meier plots for chemokine receptors ACKR1, CXCR6, CCR6, and CX3CR1, which were the only receptors that significantly correlated with the overall survival of patients with breast cancer.

**Figure 9:**
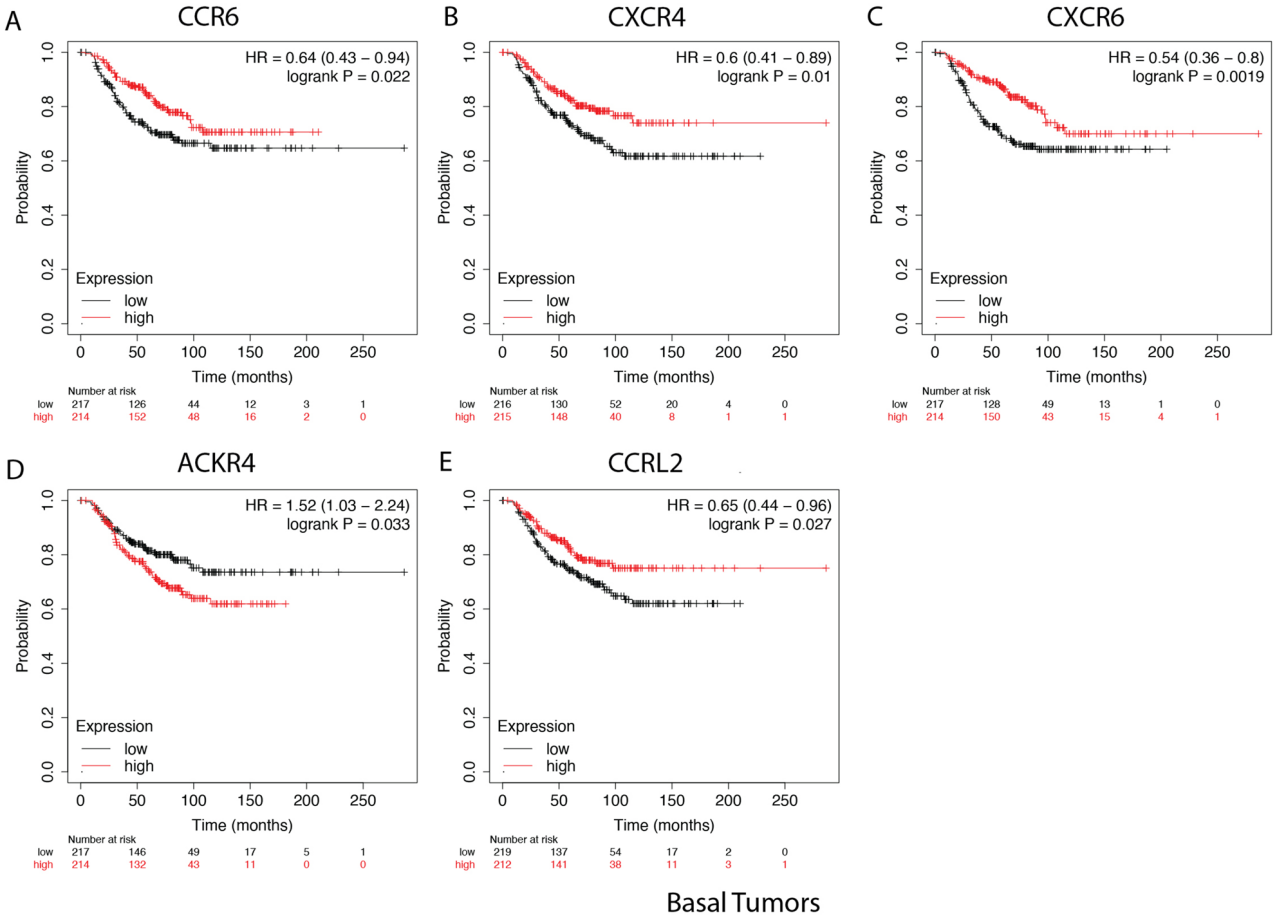
Overall survival rate of breast cancer patients tumor samples of basal subtype. Kaplan–Meier curves of overall survival based on KMplotter mRNA gene chip expression of chemokine receptors in breast cancer patients. Red line represents higher expression and black line represents lower expression. Patients with a high expression of the genes for (**A**) CCR6, (**B**) CXCR4, and (**C**) CXCR6, (**D**) ACKR4 and (**E**) CCRL2.

**Figure 10:**
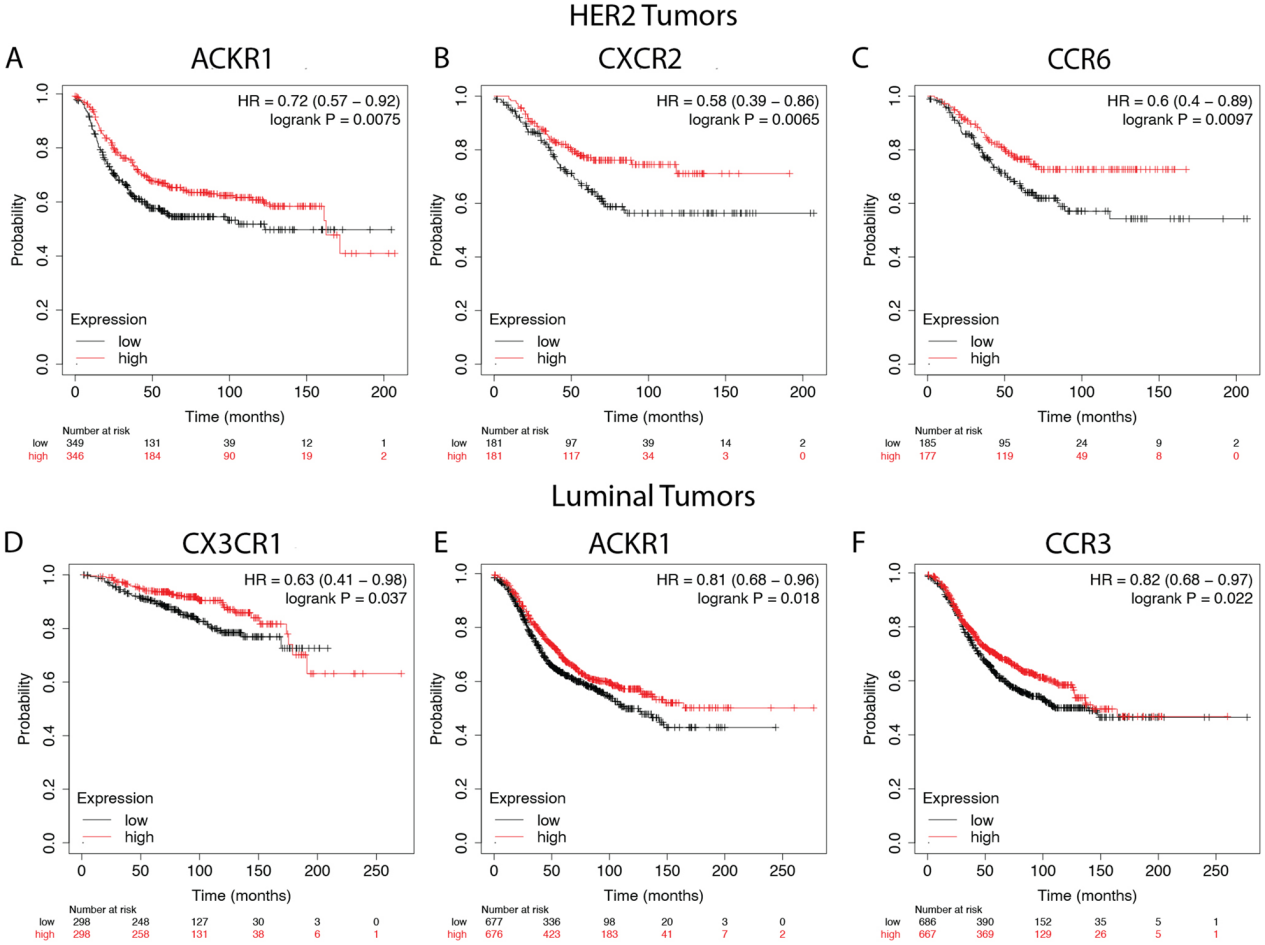
Chemokine receptors identified as significantly correlated with overall survival rate for breast cancer tumor samples of HER2 and luminal subtypes. Kaplan–Meier curves of overall survival based on KMplotter mRNA gene chip expression of chemokine receptors in breast cancer patients. Red line represents higher expression and black line represents lower expression. The receptors in (**A**) ACKR1, (**B**) CXCR2, and (**C**) CCR6, were significant for HER2 enriched subtype (**D**) CX3CR1 was correlated with the luminal A subtype. (**E**) ACKR1 and (**F**) CCR3 expression correlated with luminal B subtype.

We also assessed differences in chemokine receptors expression by race. With a p value of 0.05, higher expression of CXCR6 correlated with better overall survival for Black patients only. No significant receptor correlations with survival were observed for White or Asian breast cancer patients in the TCGA breast cancer cohort. We also looked at the Relapse-free survival rate from patients that survived with no symptoms of cancer, and found no significant differences in survival rate based on these receptor genes. Next, we assessed differences in chemokine receptors expression by molecular subtypes. Figure 9 shows the chemokine receptors that significantly correlated with breast cancer overall survival in basal tumors. Figure 10 shows the receptors that significantly correlated with overall survival rate for breast cancer tumor samples of HER2 enriched, luminal A, and luminal B subtypes.

### Chemokine receptor specific findings

#### ACKR1/DARC

Atypical Chemokine Receptor 1 or Duffy Antigen Receptor for Chemokines (ACKR1/DARC) is an internalizing receptor or chemokine-scavenging/decoy receptor whose ligand binding results in chemokine sequestration, degradation, or transcytosis because of its inability to signal via classic G-protein-mediated pathways. ACKR1 can regulate chemokine bioavailability and leukocyte recruitment when expressed on endothelial cells. It is also expressed by erythrocytes where it serves as a blood reservoir of cognate chemokines but also as a chemokine sink, buffering potential surges in plasma chemokine levels. ACKR1 can inhibit breast cancer growth and progression by sequestration of angiogenic chemokines and subsequent inhibition of tumor neovascularity^34^. DEG analysis showed that ACKR1 is significantly downregulated in Black versus White patients (Table 1), consistent with a recent study showing ACKR1 tumor expression in breast cancer is lower in African American patient’s breast cancer tumors than in European American patient tumors^22^. RNA count expression plotting for the TCGA breast cancer cohort only confirmed a significant decrease in ACKR1 expression in Asian compared to White and to Black patients (Fig. 2). In addition, there was no significant difference within the triple negative molecular subtype across the different racial categories (Fig. 4). All our different types of analyses did however, support that ACKR1 is significantly downregulated in Asian versus White and specifically in luminal A patient samples. ACKR1 expression varies significantly based on molecular subtype, with higher expression in luminal A than luminal B, HER2 enriched and triple negative breast tumors. ACKR1 associated with better overall survival (Fig. 8A).

#### CCR3

The CC Motif Chemokine Receptor 3, CCR3, is expressed in eosinophils, basophils, T, NKT, and airway epithelial cells. It may contribute to accumulation and activation of eosinophils and other inflammatory cells^35^. CCR3 has been associated with improved relapse-free survival in breast cancer with high expression of CCR3 in luminal-like rather than triple negative or HER2 enriched tumors^36^. Our results for the breast TCGA cohort show that CCR3 is higher in the triple negative subtype than in luminal A and B (Fig. 3). In addition, we found CCR3 was upregulated in luminal A, HER2 enriched, and triple negative tumors of only Black versus White patients.

#### CCR6

The CC Motif Chemokine Receptor 6, CCR6, is expressed by immature dendritic cells and memory T cells. The ligand of this receptor is macrophage inflammatory protein 3 alpha (MIP-3 alpha)^35^. CCR6 is highly expressed in pro-tumorigenic macrophages within the mammary gland microenvironment, promoting breast cancer tumors^37^. CCR6 was differentially expressed, being down regulated in Asian versus White (Table 1) and in Black versus White patients (by expression count data) (Fig. 2). High expression of CCR6 significantly associated with worse overall breast cancer patient survival (Fig. 8D), but correlated with better survival in basal and HER enriched patients (Fig. 9C, 10C). We found CCR6 is higher in luminal A and in triple negative than in luminal B breast cancers. We also found higher CCR6 expression in Black versus White patients with luminal A molecular subtype breast tumors.

#### CCRL1

The atypical chemokine receptor 4/CC‐chemokine receptor‐like 1, ACKR4/CCRL1, or CCX-CKR, is expressed by cancer cells, thymic epithelial cells, bronchial cells, and keratinocytes. ACKR4/CCRL1 down regulation correlates with worse outcome in breast cancer. CCRL1 is a negative regulator of growth and metastasis in breast cancer by sequestering chemokines and inhibiting intratumoral neovascularity^38,39^. DEG analysis shows CCRL1 was downregulated across the Black versus White patient samples in the TCGA breast cohort (Table 1). Figure 3 shows CCRL1 is significantly lower in luminal B compared to triple negative breast cancer samples. High ACKR4/CCRL1 expression correlated with worse survival in patients with triple negative or basal tumors (Fig. 9D).

#### CCRL2

The CC Motif Chemokine Receptor Like 2, CCRL2, is expressed at high levels in endothelial cells, activated macrophages, and myeloid derived leukocytes^35,40,41^. CCRL2 is a receptor for CCL19 and the chemokine/adipokine chemerin (RARRES2). CCRL2 modulates chemokine-triggered immune responses by capturing and internalizing CCL19 or presenting RARRES2 ligand to the receptor CMKLR1. CCRL2 on activated endothelial cells acts in concert with CMKLR1 to coordinate chemerin-dependent leukocyte adhesion in vitro and recruitment in vivo^41^. CCRL2 expressing adipose tissue progenitor cells cooperate to induce epithelial-to-mesenchymal transition (EMT) gene expression in luminal breast cancer cells to enhance tumor progression and metastatic dissemination^42^. This may be important in the obesity-driven inflammatory microenvironment known to correlate with tumor development and progression in post-menopausal women^42^. Our differentially expressed gene analysis shows that CCRL2 is downregulated in Black versus White patients and is upregulated in Asian versus White samples (Table 1). Figure 2 RNA count data indicates that CCRL2 is significantly upregulated in Asian versus White and Black patients. We found that CCRL2 is significantly higher in HER2 enriched than in luminal A, B, and triple negative breast tumors. CCRL2 was higher in Asian than White patients with triple negative breast cancer. CCRL2 was also higher in White than in Black patients with luminal B breast tumors. High CCRL2 correlated with better survival in triple negative or basal tumors (Fig. 9E). 

#### CXCR1 and CXCR2

Chemokine receptors CXCR1 and CXCR2 are G-protein-coupled receptors whose biological effects are mediated by the inflammatory chemokine CXCL8^43^. While not abundant in cancer cells, CXCR1 was identified on breast cancer stem-like cells (CSCs) where it plays a major role in regulating the breast CSCs and surrounding cancer cell survival via Fas-ligand mediated pathways^44,45^. Similarly, CXCR2 expression is not consistently found to be highly expressed in cancer cells within breast tumors, but rather this receptor is most highly expressed in the stromal cells of breast tumors, particularly neutrophils. High levels of CXCR2 are also associated with higher infiltration of T and B lymphocytes in breast tumors^46^. We showed that both CXCR1 and CXCR2 are downregulated in Black versus White patients based on RNA counts (Fig. 2). DEG analysis indicates downregulation of both CXCR1 and CXCR2 in the Asian versus White breast cohort (Table 1). Only CXCR2 was downregulated for both Black versus White and Asian versus White patients in the TCGA cohort (Table 1). However, DEG results for tumor only samples did not indicate significant differences for these two receptors based on race category (Table 1). Molecular subtype analysis revealed CXCR1 is significantly lower in triple negative than in luminal A, B and HER2 enriched breast cancers. CXCR2 is significantly lower in luminal B and HER2 enriched than in luminal A tumors (Fig. 3). When analyzing race category within the luminal B subtype, we found that CXCR1 was lower in Asian versus White breast tumors. Similarly, within the luminal B subtype, CXCR2 expression is lower in Black and Asian compared to White patient breast tumors (Fig. 6). CXCR2 expression correlated with better survival in HER2 enriched tumors (Fig. 10B). 

#### CXCR4

CXCR4 is a G-protein-coupled receptor highly expressed in breast cancer cells. CXCR4 plays a role in the growth and metastasis of breast cancer via its ligand CXCL12/SDF-1, which activates mTOR to promote the epithelial–mesenchymal transition (EMT), and is therefore important in metastasis^47^. CXCR4 inhibitors can impair tumor growth and metastatic dissemination in HER2 enriched breast cancer cells but do not reduce tumor growth, and can increase metastatic spread of triple negative breast cancer. CXCR4 inhibitors also reduce myofibroblast content in all breast cancer subtypes, but only decrease angiogenesis in HER2 enriched breast cancer. This is surprising considering that we would expect the more aggressive triple negative breast cancer would harbor a more angiogenic and metastatic microenvironment with increased CXCR4 expression. DEG analysis showed CXCR4 was differentially downregulated only for the Asian versus White breast cohort and samples (Table 1). Molecular subtype analysis showed CXCR4 expression was higher in triple negative samples than in luminal A, B, and HER2 enriched. CXCR4 was also higher in HER2 enriched than in luminal A and B (Fig. 3). We only found a significant difference for Asian patients with HER2 enriched, who have higher CXCR4 expression than White patients with HER2 enriched tumors (Fig. 7). CXCR4 expression correlated with better overall survival of patients with basal tumors (Fig. 9B). 

#### CXCR6

CXCR6 is a G-protein-coupled receptor enriched in inflamed tissue lymphoid cells, and also expressed in some epithelial and nonepithelial cancer cells. CXCR6 mediates tumor promoting inflammation via its ligand CXCL16 by inducing macrophage polarization toward a pro-tumoral phenotype in solid tumors^18^. Reduction of CXCR6 expression in breast cancer mouse models decreases metastasis of those tumors^48^. DEG analysis indicated CXCR6 was upregulated in the Asian versus White TCGA cohort (Table 1). Molecular subtype analysis showed CXCR6 is significantly higher in triple negative and in HER2 enriched than in luminal A and B breast tumors (Fig. 3). Higher expression of CXCR6 correlated with slightly worse overall patient survival (Fig. 8B) while CXCR6 correlated with slightly better survival for Black patients (not shown) and better survival for patients with basal tumors (Fig. 9C). This suggests that CXCR6 is potentially one of the chemokine receptors mediating pro-inflammatory microenvironment in Asian and Black breast tumors.

#### CX3CR1

The chemokine receptor CX3CR1 is over-expressed in both primary and metastatic breast tumors. CX3CR1 determines the arrest and initial lodging of breast cancer cells to transmigrate through endothelial cells and extravasate to lodge on to the skeleton in response to its ligand, Fractalkine (FKN/CX3CL1)^49,50^. Targeting CX3CR1 delays the egress of circulating tumor cells from the blood circulation, induces cancer cell apoptosis, and reduces metastasis^51^. DEG analysis shows CX3CR1 is significantly downregulated in Black versus White patient tumors (Table 1). CX3CR1 was lower in Asian patient tumor samples based on RNA gene counts (Fig. 2). Molecular subtype analysis showed CX3CR1 is significantly lower in luminal B, HER2 enriched, and triple negative breast tumors than in luminal A (Fig. 3). CX3CR1 expression associated with better overall survival (Fig. 8C)  and worse patient survival for luminal A patients (Fig. 10D).

## Discussion

We verified that expression differences observed across racial category were not confounded by molecular subtype prevalence in the different racial groups, as it is well established that minorities, such as Black women present more often with triple negative breast cancer and Asian and Latinas with HER2 enriched breast cancers than White women^8,28,29^. The expression of distinct chemokine receptors in the bulk breast cancer tissue could account for a differential recruitment of inflammatory factors such as lymphocyte infiltrate, stromal cells, and cytokine concentration gradients in the tumor microenvironment. The differential expression of these chemokine receptors across racial group category with the same molecular subtype may explain the variability in tumor microenvironment across racial groups and their potential response to future immune therapies. In addition, our analysis highlights the importance of stratifying patient racial differences within tumor molecular subtypes to better understand tumor microenvironment features. Differential gene expression showed that while there are many genes that are differentially expressed, only a few chemokine receptor genes (Table 1) were significantly different when comparing samples from White versus Black or Asian breast cancer patients. Our results warrant further studies of gene expression assessment in breast tumors across diverse patient samples to harness targeting these receptors and their distribution within cell subsets as potential therapeutics to reduce cancer disparities. A major limitation of this study is that we did not access DNA sequencing data to correlate ancestry to the patient samples’ race category, and large variations could result from incorrect or mixed self-reported racial group classification. Other limitations include the limited number of tumor samples and metastatic samples within this breast cancer cohort, as we could not associate metastatic status to expression level of our genes of interest. In addition, low sample size for minority racial groups in the TCGA makes the survival analysis by race stratification unreliable. While the TCGA is not a population-based study sample, it provides access to hundreds of patient samples with tumor genomics and clinical features making it an important dataset to form the basis of improved tumor targeted therapies, including normal tissue adjacent to the tumor samples^31^.

Despite these limitations, the chemokine receptor genes we described have been consistently identified as being involved in metastatic processes and the epithelial to mesenchymal transition, as mentioned above. In addition, here we used bulk RNA-seq findings to hypothesize about the tumor microenvironment’s chemokine receptor composition of tumor samples across race, when resolution at the single cell level will be necessary to allow us to elucidate the role of these receptor’s cellular distribution across different patient’s microenvironments.

## Methods

### Data collection

We downloaded Breast Invasive Carcinoma RNA-seq count and clinical data from The Cancer Genome Atlas (TCGA) database from 623 White, 58 Black, 52 Asian, 27 Latina and 1 American and Indian/Alaska Native patients, that had a primary tumor sample via the Genomic Data Commons (GDC) Portal on or before July 2020. Level 3 RNA-Seq data was used for this study, which is de-identified and publicly available through TCGA. The study was carried out in accordance with relevant guidelines and regulations.

### Weighted gene co-expression network analysis (WGCNA)

WGCNA was applied to the normalized, filtered, final expression matrix to identify gene groups (modules). Modules are clusters of highly interconnected genes. Only the top 33% of genes with appreciable expression levels (FPKM > 1) in more than half of the breast cancer patients were subjected to analysis. We proceeded to cut out 14 sample outliers, which resulted in 810 tumor samples for final analysis. We used the WGCNA R package^52,53^ with soft-thresholding power 6, to build a weighted gene co-expression network that contained 20,564 nodes (genes) analyzed in one block.

### Module-trait relationships

The identified modules were correlated with the available clinical traits available in the TCGA, and also the tumor molecular subtypes and the PAM50 RNA status for the available samples from the TCGA study^30^. Significant modules were plotted against clinical traits. Each row corresponds to a module eigengene, and each column corresponds to a clinical parameter, i.e. sample type, ethnicity, gender, molecular subtype, and PAM50 RNA. The module eigengene is defined as the first principal component of a given module and considered a representative of the gene expression profiles in a module. Each grid contains the correlation value, calculated based on eigengene expression and clinical traits. Listed in the heatmap are bicor correlation rho values and *p*-values for the correlation (in parentheses), defining relationships between overall weighted expression profiles of modules across samples and clinical traits. The module colors are shown on the left side of each row. The modules and number of genes each module contains are identified by a color, except for the “grey” module, which is reserved for unassigned genes with transcripts with lower correlations across samples not considered strongly co-expressed^52,53^.

### Differential expression analysis

We used DESeq2, an R package that utilizes negative binomial generalized linear models (GLM) to test for differential expression^54,55^. We analyzed the breast cancer gene expression data from the TCGA to identify genes differentially expressed in Black and Asian patients compared to White patients. We used sample expression and the associated patient metadata. The GLM used to model the effect of cancer diagnosis, stage, race, as well as other covariates such as age, on gene expression levels was: radiation_therapy + ethnicity + race. Genes differentially expressed due to race were identified by dropping the race factor from the model. Genes with false discovery rate (FDR)-corrected *p* value < 0.05 were considered significant. Common genes for normal adjacent versus tumor gene lists were identified using Venny^56^.

### Gene expression based on clinical parameters

We used RNA-seq HTSeq-counts and clinical sample data from the TCGA breast cancer patients, accessed through the GDC portal. Box and whisker plots represent the interquartile range and median line with a Log10 axis scale showing RNA-seq HTSeq-counts with global significance for ANOVA with Kruskal Wallis.

### Survival analysis

For analysis of TCGA breast cancer patients from different racial categories, we used RNA-seq HTSeq-counts and clinical sample data from the TCGA, accessed through the GDC portal. Age and race adjusted Cox KM plots were made with the survfit.coxph function in R. Expression data was divided at the median and plotted against survival time in days. For Figs. 8, 9 and 10, we used KMPlotter^57^ (http://kmplot.com/analysis/) results with logrank *p*-value < 0.05.

## Supplementary Information

Supplementary Information 1.

Supplementary Information 2.
