# Supplementary material for: Chemokine receptors differentially expressed by race category and molecular subtype in the breast cancer TCGA cohort
Source: Sci Rep. 2022 Jun 26;12:10825. doi: 10.1038/s41598-022-14734-5 (PMC9234040; doi:10.1038/s41598-022-14734-5)
Supplement: Supplementary file 1 — Supplementary Information 1. [file 41598_2022_14734_MOESM1_ESM.pdf]

# Supplementary Figure 1

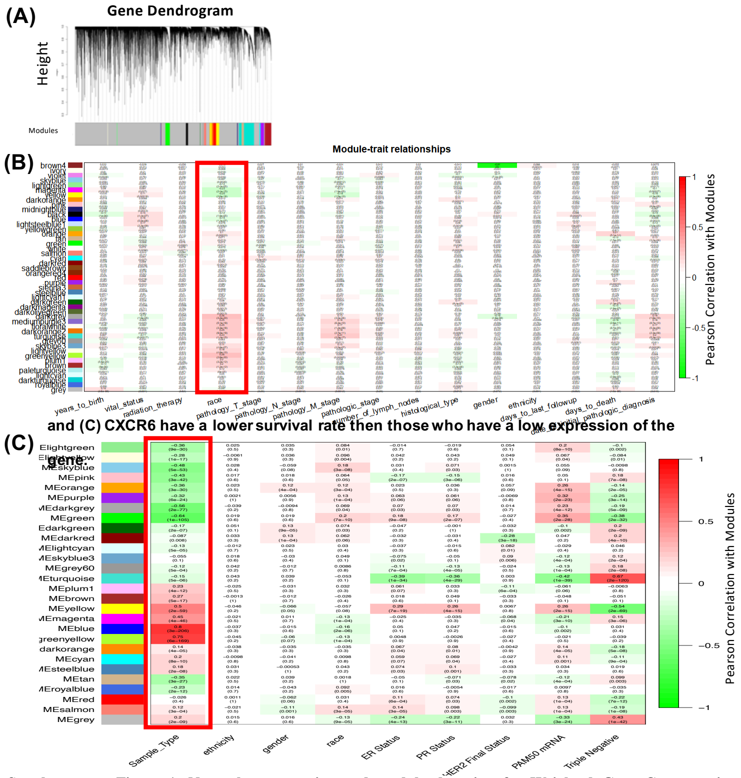


**Supplementary Figure 1. Network construction and module detection for Weighted Gene Co-expression Network Analysis (WGCNA) of Breast Invasive Carcinoma RNA-seq samples from normal and tumor samples from the TCGA database. (A)** Gene dendrogram of clustered gene dissimilarity, based on consensus topological overlap, with corresponding gene module colors of the TCGA breast invasive carcinoma RNA-seq dataset from **1202** patient samples, each **with 20253** genes all analyzed in one block. **Module-trait associations for cancer and normal samples from the TCGA (B, C).** Each row corresponds to a module eigengene, and each column corresponds to **(B)** all clinical traits available in the TCGA or **(C)** narrowed down parameters: sample type, ethnicity, gender, or molecular subtypes, and PAM50 RNA. The corresponding p-value for the correlation of the module with the clinical parameter is in parenthesis. The grid is color-coded by correlation according to the color bar of the correlation. Module-trait associations for normal and tumor samples, n = 934 samples.

# Supplementary Figure 2

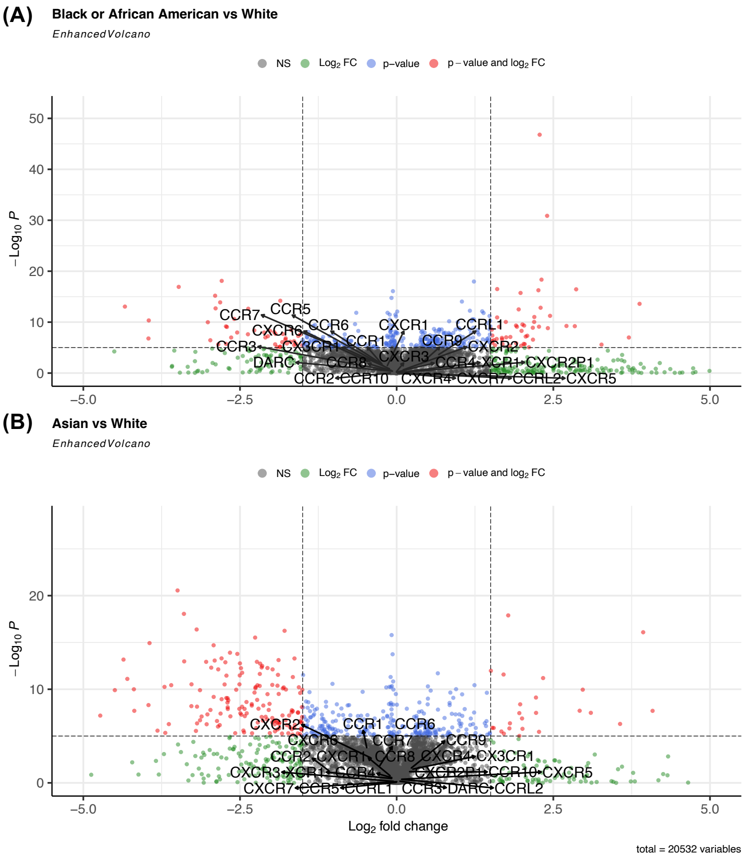


**Supplementary Figure 2. Differential Gene Expression Analysis between racial groups. (A)** Results of Differential Expression Analysis between Black or African American and White groups for tumor samples, total of 20532 genes. **(B)** Results of Differential Expression Analysis between Asian and White groups for tumor samples only.
